# Effects of Different Up-Dosing Regimens for Hymenoptera Venom Immunotherapy on Serum CTLA-4 and IL-10

**DOI:** 10.1371/journal.pone.0037980

**Published:** 2012-06-19

**Authors:** Anna Maria Riccio, Daniele Saverino, Giampaola Pesce, Anthi Rogkakou, Maurizio Severino, Patrizia Bonadonna, Erminia Ridolo, Marina Mauro, Giorgio Walter Canonica, Marcello Bagnasco, Giovanni Passalacqua

**Affiliations:** 1 Allergy and Respiratory Diseases, Department of Internal Medicine, University of Genoa, Genoa, Italy; 2 Section of Human Anatomy, Department of Experimental Medicine, University of Genoa, Genoa, Italy; 3 Medical and Radiomethabolic Therapy, Department of Internal Medicine, University of Genoa, Genoa, Italy; 4 Allergy Unit, Ospedale San Giovanni di Dio, Florence, Italy; 5 Allergy Service, Verona General Hospital, Verona, Italy; 6 Experimental and Internal Medicine, University of Parma, Parma, Italy; 7 Allergy Unit, Sant’Anna Hospital, Como, Italy; Fudan University, China

## Abstract

**Background:**

Cytotoxic T lymphocyte associated antigen-4 (CTLA-4) is involved in the activation pathways of T lymphocytes. It has been shown that the circulating form of CTLA-4 is elevated in patients with hymenoptera allergy and can be down regulated by immunotherapy.

**Objective:**

to assess the effects on CTLA-4 of venom immunotherapy, given with different induction protocols: conventional (6 weeks), rush (3 days) or ultra rush (1 day).

**Methods:**

Sera from patients with hymenoptera allergy were collected at baseline and at the end of the induction phase. CTLA-4 and IL-10 were assayed in the same samples. A subset of patients were assayed also after 12 months of VIT maintenance.

**Results:**

Ninety-four patients were studied. Of them, 50 underwent the conventional induction, 20 the rush and 24 the ultra-rush. Soluble CTLA-4 was detectable in all patients at baseline, and significantly decreased at the end of the induction, irrespective of its duration. Of note, a significant decrease of sCTLA-4 could be seen already at 24 hours. In parallel, IL-10 significantly increased at the end of the induction. At 12 months, sCTLA-4 remained low, whereas IL-10 returned to the baseline values.

**Conclusions:**

Serum CTLA4 is an early marker of the immunological effects of venom immunotherapy, and its changes persist after one year of maintenance treatment.

## Introduction

Allergy is the clinical manifestation of the “atopic status” that is characterized by an abnormal IgE response to ubiquitous substances, including pollens, foods, drugs and venoms of stinging insects. When the contact with the offending allergen is occasional and isolated (e.g. hymenoptera venom allergy, HVA, or food allergy) a “pure” IgE-mediated reaction takes place. This is essentially characterized by the sudden release of mast cell derived mediators (mainly histamine), triggered by IgE. The pure IgE-mediated diseases represent a very “clean” model to study the allergic reaction and its modulation by specific immunotherapy (SIT). T lymphocytes are responsible for the “deviation” towards the atopic status, and this deviation seems to be driven by T regulatory (Treg) cells [1], [2], so that impairment in the function of Treg cells may lead to either autoimmunity or allergy.

**Table 1:** Characteristics of patients.

	Conventional	Rush	Ultra-rush
N	50	20	24
Male/Female	38/12	11/9	20/4
Mean age (range)	48 (16–74)	45,5 (16–69)	35 (9–66)
Grade of reaction			
I	3	0	2
II	2	5	4
III	26	13	6
IV	19	2	12
VIT *Apis mellifera*	6	9	8
VIT *Polistes spp*	9	2	4
VIT *Vespula spp*	34	8	15
VIT *Vespa Crabro*	1	1	1

Many different signalling molecules intervene in the immune response, and an increasing interest is currently devoted to the activation pathways of T lymphocytes. An effective T-cell activation requires a primary signal delivered by the antigenic peptide presented by major histocompatibility complex molecules and a non-specific signal (co-stimulation) mediated by the interaction of CD28 with B7 family members (CD80, CD86) expressed on antigen-presenting cells [3]. Cytotoxic T lymphocyte-associated antigen 4 (CTLA-4), a member of the Ig superfamily, is able to bind B7 antigens, as its homologue CD28 [3]. There is evidence that CTLA-4, as distinct from CD28, is a negative regulator of T-cell activation [4]. A native soluble form of CTLA-4 (sCTLA-4), which may have immunoregulatory functions [5], [6] has also been described. High serum concentrations of sCTLA-4 have been detected in patients with various disorders, including autoimmune thyroid diseases, celiac disease and allergic asthma [6]–[8]. In addition, we have recently shown that sCTLA-4 is elevated in the serum of hymenoptera allergic patients, and decreases after venom immunotherapy (VIT) [9]. In this sense, sCTLA-4 can be regarded as a hallmark of the immunological effect of VIT.

**Figure 1:**
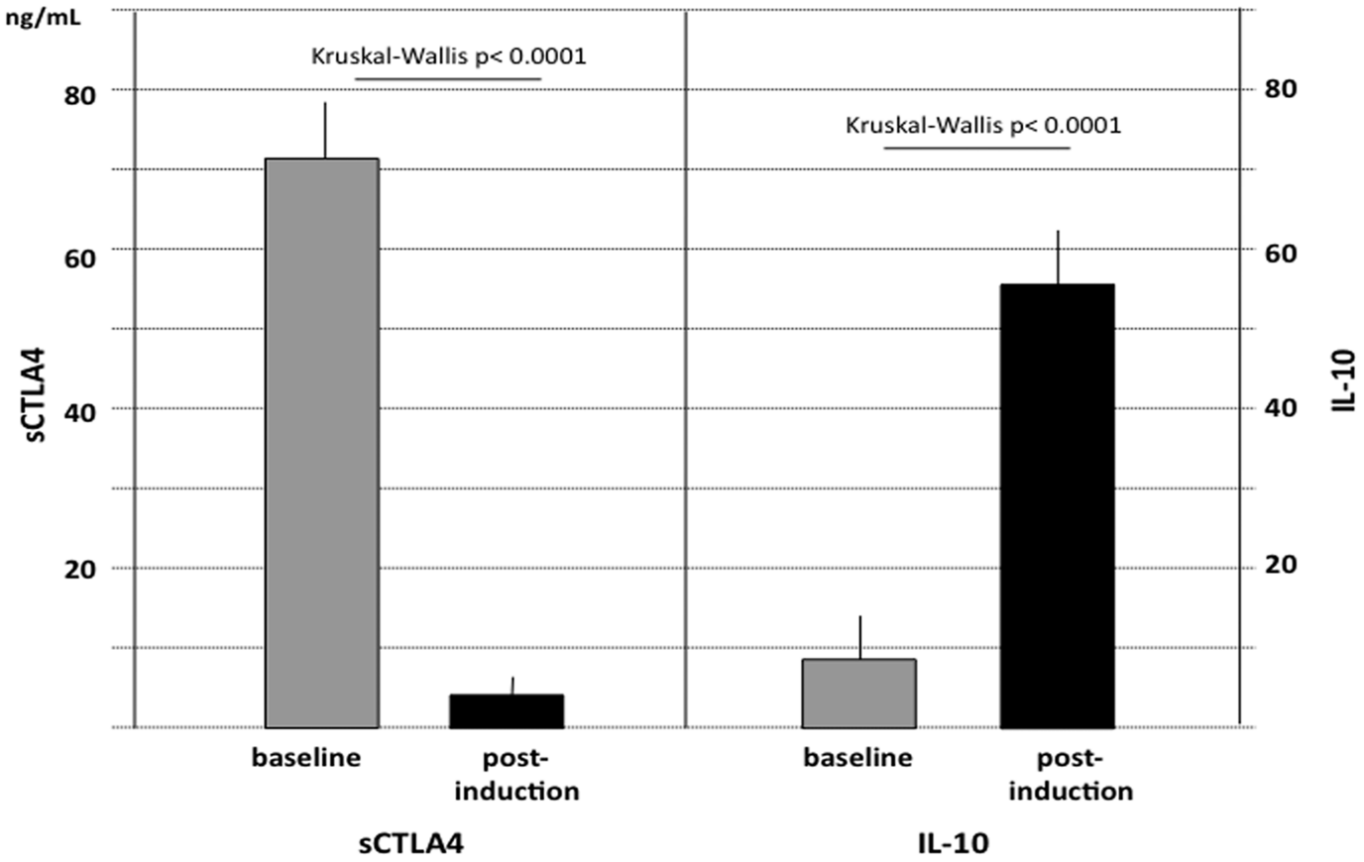
Serum CTLA4 (left) and IL-10 (right) at baseline and at the end of VIT induction phase in the whole population. Values are expressed as mean and SD. Kruskal-Wallis comparisons are also shown.

VIT can be given according to different regimens, which essentially differ in the duration of the induction phase. In the classical protocols (often defined conventional or modified rush), the build-up lasts 6 weeks or more, whereas in rush regimens the maintenance dose is achieved in 3 days or less. All those protocols are similarly effective, but there is no evidence on the timing of the immunological changes according to the induction. Based on this background, we assessed and compared the changes in sCTLA-4 induced by different regimens of VIT. Serum IL-10 was also studied in parallel.

## Methods

Patients diagnosed as having HVA, and undergoing different VIT protocols, were studied. Blood samples were collected as per routine procedures, and sCTLA-4 and IL-10 were measured at baseline and immediately after the termination of the induction phase of VIT. In a subgroup of patients, the assays were repeated after 12 months of VIT maintenance. The study is observational, and all the procedures are part of the standard clinical practice in HVA. Therefore the study was simply notified to the Ethical Committees of the participating centres, each using its approved induction protocol, according to the Italian law. The Ethical Committee approved the study. All patients (and parents in the case of minors) signed a written informed consent for the treatment of their personal data, for the diagnostic procedure and for the vaccination, according to our routine practice.

### Patients and Diagnosis

Ninety-four patients with HVA were included in this study. The diagnosis of HVA was made according to current guidelines [10], and all centres used the same diagnostic procedures. Prick tests were performed at increasing concentrations from 0.01 to 100 µg/mL, and intradermal tests involved the injection of 0.02 mL extract at 0.001 to 1 µg/mL concentrations. The tests were carried out with *Apis mellifera*, *Vespula species* (Stallergènes, Milan, Italy), *Polistes dominulus* and *Vespa crabro* (Anallergo, Florence, Italy). The Cap-RAST assay was performed with the same venoms.

**Figure 2:**
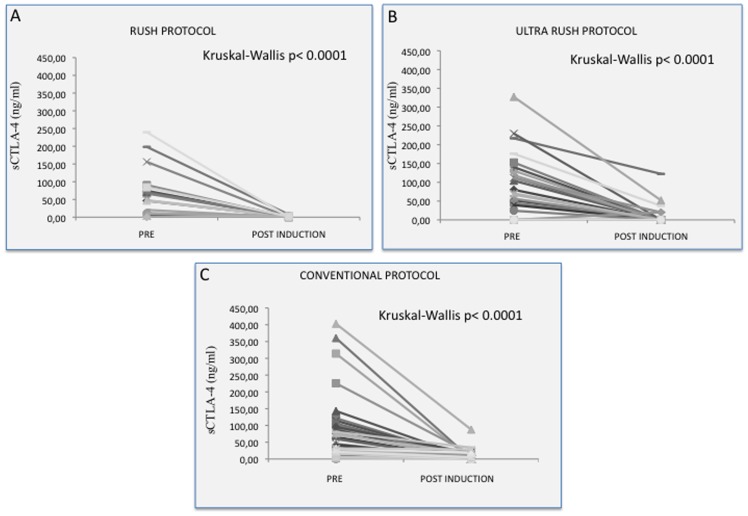
Serum CTLA4 at baseline and at the end of the induction. Three different protocols: rush (Panel A), ultra-rush (panel B) and conventional (panel C).

### VIT Protocols

The patients were prescribed with VIT for the responsible venom, but with different induction protocols as follows: a) conventional, b) rush, c) ultra-rush. The conventional induction lasted 6 weeks. On Day 1, increasing doses of 0.01, 0.1, 1 and 3 µg were administered. The subsequent doses of 10, 20, 40, 75 and 100 µg were given weekly subdivided in two injections. In the rush protocol the doses were given as follows: 0.01, 0.1, 1 and 3 µg on day 1, 5, 10 and 20 µg on day 2, 30, 35, 35 µg on day 3. Injections were given at 30-minute intervals. The ultra-rush induction lasted 210 minutes. A cumulative dose of 111.1 µg was divided into 6 injections, with an initial dose of 0.1 µg followed by 1, 10 and 20 µg at 30-minute intervals. Then 30 and 40 µg injections were given every 60 minutes. For all protocols the maintenance dose was 100 µg, given at 4–6 week intervals, and the planned duration was 3–5 years as per guidelines.

### sCTLA-4 and IL10 Assay

A blood sample was collected at the time of diagnosis (baseline), and immediately after completing the induction phase of immunotherapy. In a subset of patients, blood samples were obtained also after 12 months of maintenance VIT, and in 4 patients receiving the conventional VIT induction, sCTLA4 was measured every week from baseline to week 6. sCTLA-4 was measured by an ELISA kit (Bender Med System, Milano, Italy), according to the manufacturer’s instructions. Each sample was diluted 1∶10 and tested in triplicate. Deviation within triplicates was <10% for any reported value. The sensitivity threshold was 0.1 ng/mL. The analytical response was linear approximately between 0.162 and 1.200 of absorbance values, corresponding to 0.1–50 ng/mL (data not shown). Serum IL-10 was measured by an ELISA method (Diaclone Research, Besançon, France). The lower sensitivity threshold of the method was 5 pg/mL. Specific IgG4 were assayed by ImmunoCAP Phadia.

**Figure 3:**
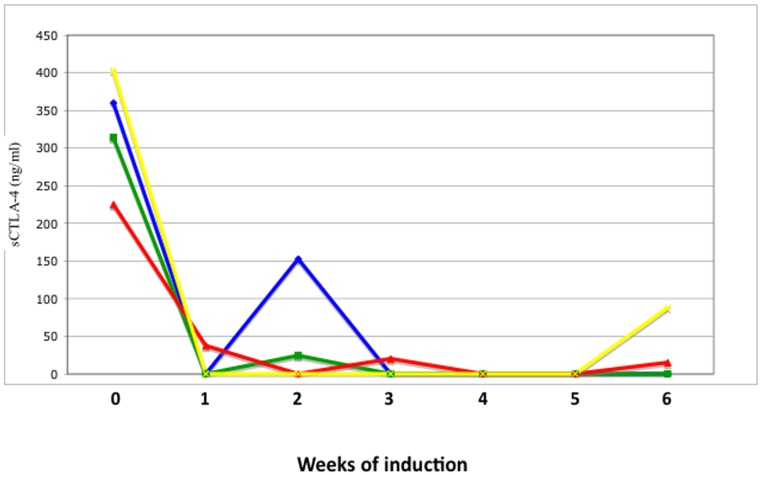
Serum CTLA4 week by week in four patients receiving the conventional 6-week induction.

**Figure 4:**
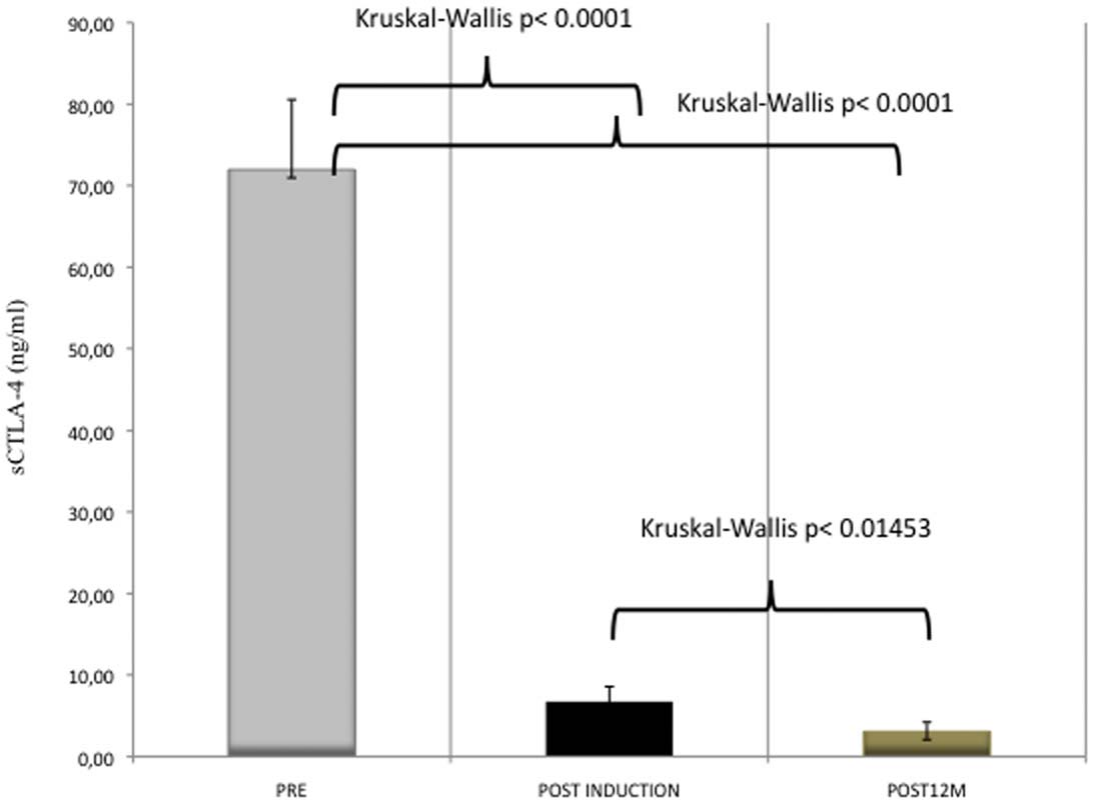
Serum CTLA4 at baseline, at the end of induction phase and after 1 year of VIT (mean, SD, and Kruskal-Wallis comparisons).

### Statistical Analysis

The Kruskall-Wallis nonparametric test was used to compare the paired samples, since a normal distribution could not be assumed. A p value <0.05 was considered significant. Nonparametric ANOVA was used to test the differences in % change before versus after induction among groups.

## Results

Ninety-four patients (69 male and 25 female, mean age 45, age range 9–74 years) were studied. None of them had a previous history of anaphylaxis due to food or drugs. Fifty subjects received the conventional induction (6 weeks), 20 the rush (3 days), and 24 the ultra-rush (24 hours) regimen. VIT was prescribed for the following venoms: 15 *Polistes*, 61 *Vespula* species, 23 *Apis Mellifera*, 3 *Vespa Crabro*. The main characteristics of the patients in the three groups are summarized in **table 1**. Out of 94 patients, 53 had one or more field re-sting, none during the induction phase. None of those 53 subjects had systemic reactions of grade II or higher, 12 had a grade I reaction and the remaining had only local reactions.

**Figure 5:**
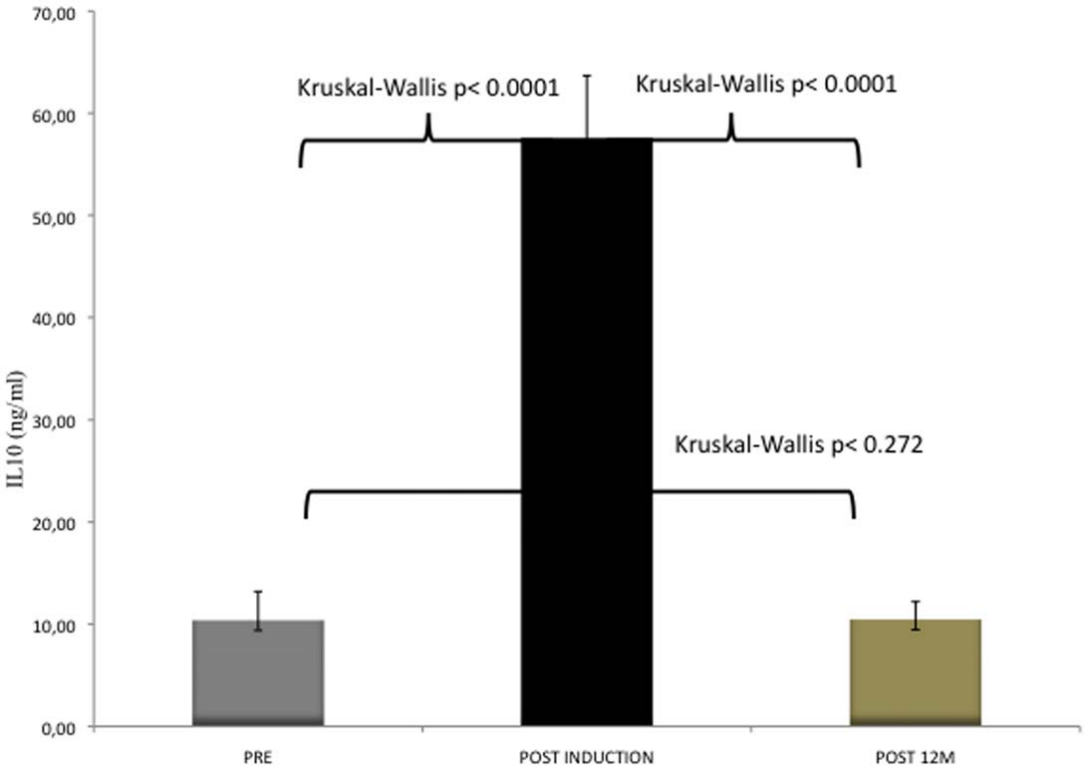
Serum IL-10 at baseline, at the end of induction phase and after 1 year of VIT (mean, SD, Kruskal-Wallis comparison).

The overall results of sCTLA4 assays before and after the induction phase of VIT are reported in **Figure 1**. According to our previous observations, all patients showed detectable levels of sCTLA4 at baseline, although distributed over a wide range of values. There was no appreciable correlation between baseline sCTLA4 and severity of the reaction. At the end of VIT induction sCTLA4 concentrations sharply decreased, irrespective of the induction protocol used, that is, independently of the duration of the induction phase (**Figure 2**). In fact, sCTLA-4 was significantly reduced as early as 24 hours also in the ultra-rush induction group (Kruskal-Wallis p<0.001). The percentage change in sCTLA4 did not differ among the three groups (one way ANOVA: R square 0.0003, p = 0.987). At the end of the induction phase the serum levels of IL-10 were significantly increased with respect to the baseline values (mean 10,39±2,82 pg/mL VS 57,58±6,10 pg/mL; p<0.0001) (**Figure 1**). Also in this case, the increase was apparent at the end of the induction phase whatever the induction protocol (not shown). The percentage change in IL-10 did not differ among the three groups (one way ANOVA: R square 0.025, p = 0.3427). In the four patients who had sCTLA4 measured week by week, a sharp decrease of sCTLA-4 could be observed starting from the first week (**Figure 3**).

Both sCTLA-4 and IL-10 were measured after 12 months of maintenance VIT in 69 patients. The concentration of sCTLA-4 remained lower than at baseline at post-induction, with a further significant decrease at 12 months of maintenance (Kruskal-Wallis p = 0.015) (**Figure 4**). On the contrary, the concentration of IL-10 at 12 months returned to baseline levels (mean 10,46±1,74 VS 10,39±2,82 p = ns) (**Figure 5**).

## Discussion

CTLA-4 is recognized as a negative regulator of T-cell activation. In fact, the binding of CTLA-4 to the T-cell surface initiates a cascade of biochemical events that attenuates the ongoing immune response [6]. The most convincing data supporting such a role for CTLA-4 come from experiments in knockout mice where the *CTLA-4* gene is inactivated [11]. These mice display severe polyclonal lymphoproliferative disorders that infiltrate most organs, and die in a few weeks. CTLA-4 may play a role also in atopy, where the activation of T cells is crucial for the regulation of the immune response [12], [13]. Since CTLA-4 can be present as a native soluble form in the serum, and sCTLA-4 is able to bind its ligands and displays functional activities [5], [6], we previously assessed its behaviour in a pure IgE mediated disease such as HVA [9]. We showed that sCTLA-4 was increased in all patients with HVA, but not in respiratory allergy or in healthy subjects. Of note, the role of sCTLA-4 is not well ascertained in respiratory allergy, as demonstrated by controversial results [14]–[16]. On the other hand, CTLA-4 appears to be consistently elevated in HVA and sharply decreased after the induction phase of VIT. This reduction parallels the increase in IL-10 [9]. As such, serum sCTLA-4 seems to discriminate patients with HVA with respect to healthy subjects and patients with allergic respiratory diseases. In the present study we aimed at assessing if the duration of the VIT induction phase influences the behavior of circulating sCTLA-4, and if there are modifications on the long-term. We observed that the decrease in CTLA-4 is a very early phenomenon that occurs immediately after induction whatever the induction protocol: the faster was the induction, the earlier sCTLA4 decrease. The magnitude of decrease seems to be merely related to the amount of allergen administered, without any relationship with induction timing. Long-term follow-up demonstrated that sCTLA4 concentrations remained low as long as 12 months after starting VIT. Of note, the changes in IL-10 are very early, within 24 hours. On the other hand IL-10 returned to baseline level after one year, thus suggesting that this cytokine intervenes only in the early phases of specific immunotherapy [17], whereas sCTLA4 testifies for the persistence of the immunologic effects of vaccination.

Thus, although the two phenomena (sCTLA4 decrease and IL10 rise) occur abruptly and nearly simultaneously, they are not strictly dependent between each other, in other words, sustained IL10 secretion is not required to maintain low sCTLA4 levels in serum. This is perhaps not surprising, since it is well conceivable that other cytokines/regulatory signals play some role. It is worth noting, in addition, that IL10 secretion following VIT is likely due to different cell types (T-cells and monocytes) with different timing and kinetics [18], [19]. In any case, we hypothesize that sCTLA4 reduction may reflect subsiding of sustained T-cell activation in patients under VIT maintenance. It is conceivable that production of sCTLA4 primarily depends upon T- cell activation (irrespective to their specificity or cytokine secretion capability), and that sCTLA4, which proved to be a functional molecule, plays a role in perpetuating the activation state of T-cells by interfering with negative signalling through membrane CTLA4 [6]. Thus, VIT, that is expected to redirect the immune response to the allergen(s), results in stable reduction of sustained activation state of T-cells. Of note, it is possible that sCTLA4 also derives from cell types other than T-lymphocytes; in addition and more importantly, as previously discussed [6] the interaction of sCTLA4 with resting T-cells may act in an inhibitory way, by interfering with stimulatory signalling through membrane CD28 expressed by resting T-cells.

In conclusion, serum sCTLA4 reduction following VIT induction, whatever the protocol used, is a very early marker of VIT effectiveness, and it is possible to envisage that the persistence of low sCTLA4 levels indicates sustained protection against clinical reactions to field-resting.
